# Metabolism in Invariant Natural Killer T Cells: An Overview

**DOI:** 10.20900/immunometab20210010

**Published:** 2021-02-10

**Authors:** Emily L. Yarosz, Cheong-Hee Chang, Ajay Kumar

**Affiliations:** 1 Immunology Graduate Program, University of Michigan Medical School, Ann Arbor, MI 48109, USA; 2 Department of Microbiology and Immunology, University of Michigan Medical School, Ann Arbor, MI 48109, USA

**Keywords:** iNKT cells, CD4 T cells, metabolism, glycolysis, OXPHOS, fatty acid synthesis, cell proliferation

## Abstract

Cellular metabolism is critical for generating energy and macromolecules for cell growth and survival. In recent years, the importance of metabolism in mediating T cell differentiation, proliferation, and function has been a hot topic of investigation. However, very little is known about metabolic regulation in invariant natural killer T (iNKT) cells. In this viewpoint, we will discuss what is currently known about immunometabolism in iNKT cells and how these findings relate to CD4 T cells.

## INTRODUCTION

The study of immunometabolism has significantly advanced our understanding of T cell biology. Resting T cells in the periphery maintain quiescence by utilizing primarily oxidative phosphorylation [1]. In contrast, activated T cells undergo a metabolic switch from an oxidative to a glycolytic phenotype in order to generate high levels of ATP and other macromolecules for their rapid proliferation [1]. After resolution of an immune response, activated T cells either die or transition into memory cells, which rely mainly on fatty acid oxidation (FAO) for their maintenance [2]. The different T helper cell subsets also operate distinct metabolic programs, which contribute to the development of subset-specific functions. For example, Th1, Th2, and Th17 cells all perform aerobic glycolysis to support their function [2]. However, both Th1 and Th17 cells display high levels of mammalian target of rapamycin complex 1 (mTORC1) activity while Th2 cells display high levels of mammalian target of rapamycin complex 2 (mTORC2) activity [3]. Like memory CD4 T cells, regulatory T cells perform FAO for their survival and metabolic needs [2,3]. Despite our comprehensive knowledge of conventional T cell metabolism, little is known about natural killer T cell metabolism.

Natural killer T cells are innate-like T cells that recognize glycolipid antigens in the context of the MHC class I-like molecule CD1d [4]. There are two major categories of natural killer T cells: invariant natural killer T (iNKT) cells and type II natural killer T cells. The development of both types of natural killer T cells depends on the expression of the lineage defining transcription factor promyelocytic leukemia zinc finger (PLZF) [5,6]. However, the two types of cells differ in the makeup of their T cell receptors (TCR). iNKT cells express a semi-invariant αβ TCR, while type II natural killer T cells express diverse αβ TCRs [7–10]. Because type II natural killer T cells are present in low frequencies in murine model systems, much of what we know about natural killer T cell metabolism is derived from iNKT cells. In this viewpoint, we will highlight what is currently known about immunometabolism in iNKT cells and what the future holds for this exciting field.

## iNKT CELLS HARBOR BOTH INNATE-LIKE AND ADAPTIVE FUNCTIONS

As part of the T cell lineage, iNKT cells undergo stage-wise development in the thymus. iNKT cells are positively selected on cortical thymocytes expressing CD1d during the DP stage of development [11]. Following positive selection, iNKT cells mature through a series of stages that are classified by CD24, NK1.1, and CD44 expression: CD24^+^ NK1.1^−^ CD44^−^ stage 0 cells, CD24^−^ NK1.1^−^ CD44^−^ stage 1 cells, CD24^−^ NK1.1^+^ CD44^−^ stage 2 cells, and CD24^−^ NK1.1^+^ CD44^+^ stage 3 cells [12–14]. Of these stages, stage 0 and stage 1 cells are the most immature and have a highly proliferative phenotype, whereas stage 3 iNKT cells are the most mature and have a quiescent phenotype [12]. Additionally, iNKT cell subsets have been reported in the thymus. These subsets are termed iNKT1, iNKT2, and iNKT17 cells and are classified by the expression of the transcription factors T-bet, GATA-3, and RORγt, respectively [11]. Like the conventional T helper cell subsets, thymic iNKT cell subsets display distinct cytokine profiles, with iNKT1 cells producing high levels of IFNγ, iNKT2 cells producing high levels of IL-4, and iNKT17 cells producing high levels of IL-17 [11].

Mature iNKT cells exit the thymus and populate the peripheral tissues as effector cells capable of rapidly responding to antigen. The liver is home to a large depot of iNKT cells, with IFNγ-producing iNKT1 cells making up 40% of all T cells in this organ [15]. iNKT1 cells are also enriched in the spleen, but these cells are balanced by the presence of iNKT2 and iNKT17 cells [15]. The lungs, intestines, and lymph nodes exhibit a similar iNKT cell composition to the spleen [15]. Some iNKT cells produce high levels of IL-10 [16], imparting a regulatory phenotype upon this group of cells. Regulatory iNKT cells are often termed iNKT10 cells, and these cells typically seed the adipose tissue. iNKT10 cells maintain adipose tissue homeostasis by clearing apoptotic adipocytes from fat depots, controlling adipose tissue-resident T cell and macrophage populations, and promoting an anti-inflammatory tissue microenvironment [15,17–19]. However, iNKT10 cells can also mediate harmful effects in the context of obesity and other metabolic syndromes [17]. In all, iNKT cells maintain tissue homeostasis by acting as a lymphoid bridge between the innate and adaptive immune systems.

## NUTRIENT-SENSING IMMUNE SIGNALING PATHWAYS DIRECT iNKT CELL DEVELOPMENT AND EFFECTOR FUNCTION

mTOR is a conserved serine/threonine kinase that plays a central role in controlling nutrient sensing and cell growth. The mTOR signaling pathway also regulates metabolic programming in T cells [2,3]. The mTOR signaling pathway is comprised of two major signaling complexes termed mTORC1 and mTORC2. Increased import of amino acids and glucose after T cell activation triggers mTORC1 activation, while phosphoinositide 3-kinase (PI3K) signaling downstream of the TCR triggers mTORC2 activation [2]. mTORC1 and mTORC2 contain the scaffolding proteins regulatory-associated protein of mTOR (Raptor) and rapamycin-insensitive companion of mTOR (Rictor), respectively [2]. Mice having T cell-specific deletions of either Raptor or Rictor exhibit a severe block in iNKT cell development [20,21]. Raptor deficient iNKT cells not only accumulate in stages 0 and 1 but are also biased towards iNKT2 differentiation [21]. In contrast, deletion of Rictor leads to decreased stage 1 iNKT cell proliferation, decreased thymic iNKT cell IL-4 and IL-17 production, and decreased iNKT2 and iNKT17 differentiation [20,22]. Beyond Raptor and Rictor, the mTOR signaling inhibitor Tsc1 is also important for iNKT cell development, as iNKT cells lacking Tsc1 accumulate in stage 2 [23]. The transcription factor cellular myelocytomatosis oncogene (c-Myc), which is downstream of mTORC1, has also been shown to be a critical regulator of proliferation in early stage iNKT cell development [24]. However, mTOR activation must be balanced over the course of iNKT cell development, as persistent mTOR activity inhibits iNKT cell development and leads to iNKT cell death [25]. Like developing iNKT cells, mature iNKT cells upregulate mTORC1 and mTORC2 activity after stimulation [26]. Inhibition of mTOR during iNKT cell activation not only blocks iNKT cell growth and proliferation but also dampens inflammatory cytokine production [26]. In all, mTOR activation in both thymic and peripheral iNKT cells is critical to ensure proper iNKT cell development and acquisition of effector functions.

The conserved Hippo signaling pathway has also been identified as a regulator of iNKT cell development and metabolism. Environmental cues such as cell-to-cell contact activate the conserved Hippo signaling pathway, which blocks cell proliferation by inhibiting the transcription factors Yes-associated protein (YAP) and transcriptional coactivator with PDZ motif (TAZ) [27]. There are two integral serine/threonine kinases in the mammalian Hippo signaling pathway: Mst1 and Mst2 [27]. Mst1 promotes efficient adhesion of lymphocytes to the high endothelial venules, allowing for proper lymphocyte trafficking to lymphoid organs [28]. Recently, Mst1 and Mst2 have been shown to be critical for iNKT cell development and survival [29]. Loss of Mst1 leads to increased iNKT17 differentiation in the thymus via uncontrolled ICOS-mTORC2 signaling in developing iNKT cells [29]. Mst1 deficient iNKT cells also show aberrant mitochondrial homeostasis in stage 3 of iNKT cell development, preventing mature iNKT cells from acquiring a quiescent metabolic phenotype [29]. Taken together, recent studies focused on the Hippo signaling pathway provide further evidence for careful communication between cellular metabolic and immune signaling pathways for proper iNKT cell development and function.

## GLUCOSE METABOLISM HAS CONTEXT-DEPENDENT EFFECTS ON iNKT CELL PROLIFERATION AND FUNCTION

Conventional T cells rely on glucose as a major source of energy [2]. Glucose is catabolized through two main pathways: glycolysis and the pentose phosphate pathway (PPP). During glycolysis, glucose is broken down into pyruvate [30]. Pyruvate is then converted into either lactate or acetyl-CoA, which is oxidized in the mitochondria by the tricarboxylic acid (TCA) cycle [30]. The TCA cycle also generates redox reagents that participate in the electron transport chain along the inner mitochondrial membrane, which results in ATP production [30]. Electron transport chain activity is commonly known as oxidative phosphorylation (OXPHOS). During the PPP, glucose is rapidly broken down to produce both nucleotide precursors and NADPH molecules [30]. NADPH plays a critical role in lipid biosynthesis and other anabolic reactions necessary for cell growth [30].

Developing iNKT cells appear to use glucose for proliferation, whereas peripheral iNKT cells use glucose to support their effector function. Immature iNKT cells have higher glucose uptake and higher expression of the glucose transporter Glut1 compared to mature iNKT cells in the thymus [31]. This heightened reliance on glucose is thought to fuel the rapid proliferation of stage 0 and stage 1 iNKT cells [31]. However, little is known about how mature iNKT cells utilize glucose. Using a whole metabolomics approach, our lab recently showed that quiescent iNKT cells harbor higher levels of TCA cycle metabolites than quiescent CD4 T cells do [26]. In line with this, activated iNKT cells in the periphery upregulate glucose uptake in comparison to quiescent iNKT cells [26]. iNKT cell stimulation also increases expression of genes encoding metabolic enzymes involved in the PPP and the TCA cycle [26]. These results suggest that activated iNKT cells use glucose mainly to fuel the PPP and OXPHOS. Additionally, limiting glucose during mature iNKT cell activation does not affect iNKT cell survival or proliferation but leads to a severe defect in the production of IFNγ, IL-4, and IL-17 [26]. Our lab has also shown that glucose metabolism is important for peripheral iNKT cell responses against pathogens, as increased glucose uptake correlates with increased IFNγ production by activated iNKT cells and enhanced clearance of *Listeria monocytogenes* infection [26].

## iNKT CELL DEVELOPMENT AND FUNCTION RELIES ON THE TIGHT CONTROL OF REACTIVE OXYGEN SPECIES

Our lab found that resting iNKT cells maintain high intracellular levels of reactive oxygen species (ROS) [32], byproducts of aerobic metabolism and other pathways that can be toxic to the cell. These ROS are crucial for the development of inflammatory functions in peripheral iNKT cells and skew the iNKT cell response toward iNKT1 and iNKT17 [32]. High basal levels of ROS are generated by NADPH oxidases in iNKT cells, and PLZF regulates ROS production in iNKT cells [32]. However, the mechanisms by which iNKT cells maintain cellular redox balance to avoid toxicity and death remain unclear.

The Nrf2-Keap1-Cul3 trimeric complex is a major regulator of redox balance in mammalian cells. Under homeostatic conditions, the BTB-domain-containing adaptor protein Keap1 binds to the transcription factor Nrf2 [33], allowing the E3 ubiquitin ligase Cul3 to ubiquitinate Nrf2. This ubiquitination targets Nrf2 for proteasomal degradation [34]. Under times of oxidative stress, the trimeric complex dissociates, allowing Nrf2 to translocate into the nucleus and activate antioxidant response element (ARE)-containing genes [35,36]. These ARE-containing genes lead to the production of antioxidants to combat rising ROS levels.

Recently, our lab has shown that the Nrf2-Keap1-Cul3 trimeric complex is critical for iNKT cell homeostasis. Mice having a T cell-specific deletion of Keap1 display aberrant iNKT cell development in the thymus [37]. Additionally, Keap1 deficient iNKT cells exhibit lower total ROS levels but higher glucose uptake, glucose transporter expression, and mitochondrial function compared to wild type iNKT cells in the periphery [37]. These phenotypes are due to increased levels of Nrf2 in the absence of Keap1 [37], indicating that high levels of Nrf2 may be detrimental to both developing and peripheral iNKT cells. However, more work is necessary to uncover the role of Nrf2 in iNKT cell homeostasis.

Because the ubiquitin ligase Cul3 is also part of the Nrf2-Keap1-Cul3 trimeric complex, we believe that Cul3 may also control metabolic programming in iNKT cells. Cul3 is essential for iNKT cell development, as iNKT cells lacking Cul3 fail to mature and acquire an effector phenotype [38]. Cul3 is also known to colocalize with PLZF in the nucleus of mature iNKT cells [38]. Although the exact metabolic targets of PLZF remain unknown, our lab has shown that PLZF inhibits both glycolysis and mitochondrial function in iNKT cells [26]. However, the impact of Cul3 on iNKT cell metabolism has not been tested. The interaction between Cul3 and PLZF raises the interesting possibility that Cul3 may use PLZF as a transport protein to reach the nucleus. Once in the nucleus, Cul3 may modulate the expression of metabolic genes and enzymes, as Cul3 is known to interact with several epigenetic modifiers [38].

iNKT cells also rely on autophagy to control ROS levels and prevent cellular damage during development. Loss of the autophagy-related genes Atg5 and Atg7 leads to iNKT cell developmental arrest during the early stages of development [31,39]. Autophagy has also been shown to be a key regulator of cell cycle progression in thymic iNKT cells [39]. Mitophagy, a specialized form of autophagy dedicated to the breakdown of mitochondria, regulates iNKT cell mitochondrial mass and mitochondrial reactive oxygen species (mROS) production as the cells progress through development [31]. In fact, iNKT cells lacking Atg7 show increased mitochondrial content and mROS production compared to wild type cells [31], leading to increased rates of apoptosis in autophagy deficient iNKT cells [31,39]. Although the role of autophagy in peripheral iNKT cell homeostasis and function remains unknown, autophagy seems to inhibit mitochondrial metabolism during iNKT cell development.

## LIPID METABOLISM DAMPENS INFLAMMATORY iNKT CELL RESPONSES

In addition to glucose, lipids can also be metabolized in order to influence T cell differentiation and function. Increased activity of acetyl Co-A carboxylase, an enzyme crucial for regulating fatty acid metabolism, favors regulatory T cell development and inhibits differentiation of Th17 cells [40]. Moreover, development of memory CD8 T cells requires lipolysis to support fatty acid catabolism through β-oxidation [41].

Lipid synthesis has recently emerged as a critical regulator of iNKT cell responses. Interestingly, β-oxidation does not influence iNKT cell function [42]. However, iNKT cells have been shown to harbor higher levels of PPARγ, a regulator of lipid metabolism, than CD4 and CD8 T cells [42]. Additionally, activated iNKT cells increase cholesterol synthesis to promote their proliferation and cytokine production. Inhibition of cholesterol synthesis reduces TCR signaling and IFNγ production by activated iNKT cells. In contrast, IL-4 production by iNKT cells remains largely intact in the absence of cholesterol synthesis, as glucose appears to be more important for IL-4 production in iNKT cells [26,42]. The impact of lipid biosynthesis on iNKT cell function is further highlighted by the fact that polarization of iNKT cells towards an iNKT1 phenotype results in decreased tumor growth and increased survival of tumor-bearing mice [42]. Therefore, therapeutics that skew iNKT cell metabolism towards lipid biosynthesis may have the potential to increase iNKT cell inflammatory function and protect against tumor progression.

Although de novo fatty acid synthesis appears to support iNKT cell inflammatory function, high levels of extracellular lipid may suppress inflammatory cytokine production by iNKT cells. Treatment with exogenous palmitic acid, a long chain fatty acid, induces the unfolded protein stress response in the endoplasmic reticulum and leads to the degradation of mRNA encoding the transcription factors T-bet and GATA-3 in expanded iNKT cell lines [43]. These transcription factors are crucial for the differentiation of iNKT1 and iNKT2 subsets, respectively. As such, palmitic acid inhibits IFNγ and IL-4 production by activated iNKT cells [43]. Additionally, mice fed a palmitic acid-rich diet exhibited less joint inflammation in a model of rheumatoid arthritis than mice fed a control diet [43]. These findings suggest that a diet rich in long chain fatty acids may be beneficial for patients with inflammatory autoimmune diseases. Together, lipid metabolism may have context-dependent effects on iNKT cell inflammatory function, as evidenced by the opposing effects of intracellular and extracellular lipids on iNKT cells in human disease.

## iNKT CELLS AND CD4 T CELLS OPERATE DISTINCT METABOLIC PROGRAMS

**Figure 1.**
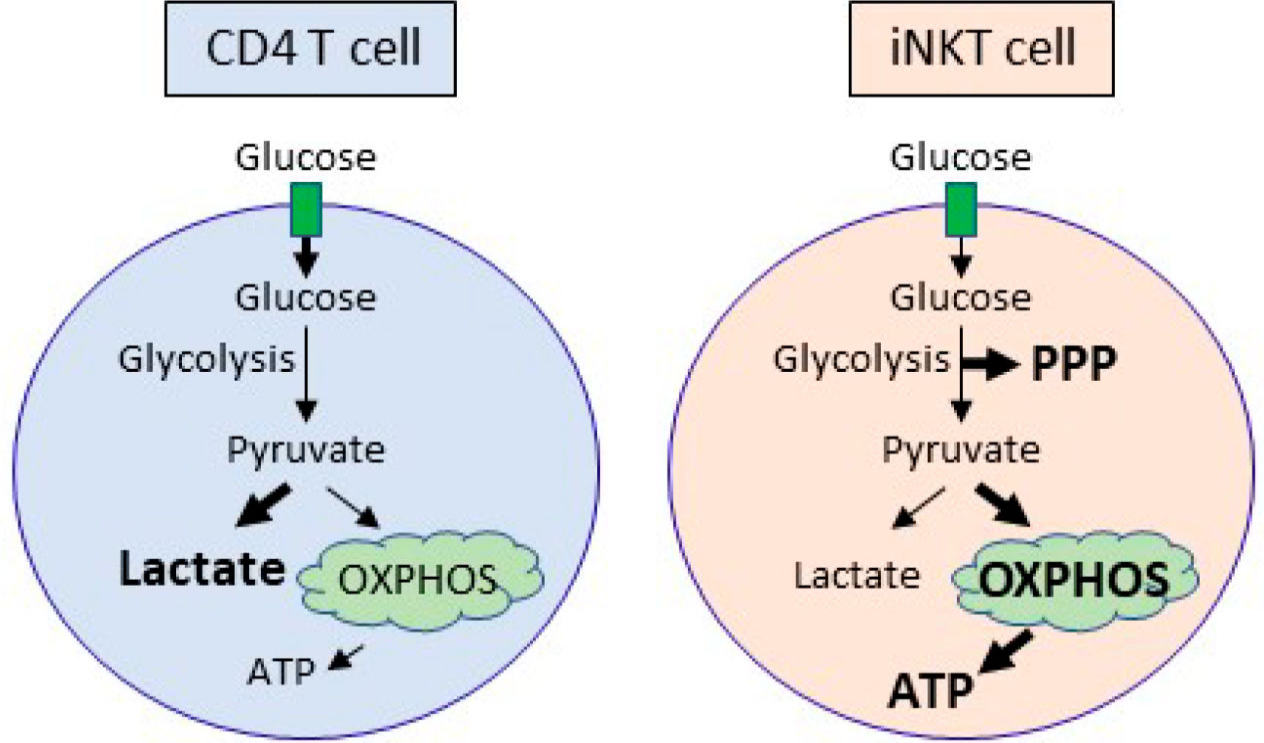
Glucose is differentially utilized in CD4 T cells and iNKT cells after stimulation. Activated CD4 T cells metabolize glucose primarily into lactate, whereas iNKT cells support the PPP and OXPHOS. Activated iNKT cells produce more ATP than CD4 T cells, which is likely due to elevated OXPHOS in iNKT cells. Abbreviations: ATP, adenosine triphosphate; PPP, pentose phosphate pathway; OXPHOS, oxidative phosphorylation.

iNKT and conventional CD4 T cells prioritize the use of different metabolic pathways to mediate their cell type-specific functions (Figure 1). CD4 T cells primarily metabolize glucose into lactate via glycolysis, whereas iNKT cells metabolize glucose via the PPP and OXPHOS. In line with this observation, iNKT cells harbor more ATP than CD4 cells both before and after activation [26]. Peripheral iNKT cells also have lower glucose uptake compared to conventional CD4 T cells, as PLZF inhibits glycolysis [26]. Although both CD4 and iNKT cells need glutamine for their proliferation, optimal expansion requires glucose in CD4 T cells and fatty acid metabolism in iNKT cells. Similar to Th1 and Th17 cell subsets [3], iNKT cells also require glucose to produce IFNγ, IL-4, and IL-17. However, whether the different iNKT cell subsets have unique metabolic requirements is an area of open investigation.

## iNKT CELL METABOLISM: WHAT’S NEXT?

Little is known about how nutrient availability in the tissue microenvironment impacts metabolic programming in tissue-resident iNKT cell populations. iNKT cells make up 40% of all T cells located in the liver and 20% of all T cells located in the adipose tissue [15]. These cells must adapt to inflammatory and nutrient-deprived environments to maintain proper host defense. Accumulating data suggest that the microenvironment can have a profound effect on immune cell function [44]. High rates of OXPHOS appear to be important for ensuring iNKT cell survival, as high levels of lactate in the cellular microenvironment lead to increased iNKT cell death [26]. Additionally, lactic acid supplementation during iNKT cell culture inhibits activation of the nutrient sensor mTORC1, reducing iNKT cell proliferation and cytokine production [26]. This block in mTORC1 signaling in the presence of high environmental levels of lactic acid may be due to impaired cholesterol synthesis [42,45]. However, dissecting the role of tissue environment on iNKT cell metabolism can be challenging, as in vitro cell culture conditions are known to be quite different from the in vivo microenvironment [46]. Additionally, it is not known how metabolite availability differs in the various bodily tissues. As such, hepatic iNKT cells may exhibit differences in metabolism compared to splenic and adipose tissue-resident iNKT cells. Furthermore, iNKT cells present in the adipose tissue do not express PLZF [18]. Because PLZF inhibits glycolysis [26], adipose tissue-resident iNKT cells are likely more glycolytic than other tissue-resident iNKT cells. As such, more work is needed to uncover the metabolic requirements of different tissue-resident iNKT cell pools.

Although the need for glucose and fatty acids in iNKT cells is beginning to become clear, the role of amino acids like glutamine in regulating iNKT cell responses remains unknown. During glutaminolysis, glutamine is converted to glutamate [47]. Interestingly, iNKT cells cultured in the absence of glutamine show a marked defect in proliferative capacity [26], suggesting that glutaminolysis may play a key role in regulating activation-induced iNKT cell expansion. Glutamine also appears to be important in supporting iNKT cell survival, as iNKT cells grown in the absence of glutamine have increased rates of cell death compared to iNKT cells grown in glutamine-replete conditions [26]. Beyond glutaminolysis, glutamine also serves as a precursor for synthesizing proteins and nucleotides [47]. As such, how glutamine governs iNKT cell responses begs further experimentation.

## CONCLUSION

Immunometabolism is an exciting area of research in the field of immunology, having significant therapeutic potential to improve human health. While the field has made tremendous progress in understanding how metabolic pathways regulate conventional T cell function and differentiation, continued work is needed to fully unravel the role of metabolism in iNKT cells. For instance, little is known about metabolic regulation during iNKT cell development. Additionally, it is unclear whether the iNKT cell subsets operate distinct metabolic programs for their function, similar to what is seen in conventional T helper cell subsets. Metabolite availability in various tissues raises another layer of complexity, and tissue-resident iNKT cell subsets may show a preference for metabolic pathways that are not observed in circulating iNKT cells. These questions highlight important gaps in our knowledge of iNKT cell metabolism.

Exploring how metabolism drives iNKT cell proliferation, function, and survival may provide new therapeutic targets to improve T cell-based therapies in the future. Although iNKT cells constitute only a fraction of the overall T cell population in the human body, they exert critical and non-redundant functions in disease states like cancer and infection [48]. iNKT cell adoptive transfer has recently emerged as a safe and effective treatment for cancer patients [49–53]. In order to grow enough cells for adoptive transfer, iNKT cells are typically expanded in vitro and activated with cytokines or the synthetic glycolipid α-galactosylceramide. However, the findings outlined in this review show that manipulating iNKT cell metabolism can increase iNKT cell inflammatory function. Therefore, growing iNKT cells in culture conditions that skew iNKT cell metabolism to favor iNKT1 differentiation may also be an effective method of priming iNKT cells for adoptive transfer into patients. In all, the manipulation of iNKT cell metabolism has tremendous therapeutic potential, and a better understanding of how metabolism is regulated in iNKT cells is necessary to fully realize this potential.
